# Knowledge, attitudes, and practices towards Kawasaki disease from caregivers of children with Kawasaki disease: a cross-sectional study

**DOI:** 10.1186/s12889-024-18407-y

**Published:** 2024-03-26

**Authors:** Miaomiao Zhao, Jiaxin Ye, Luping Chen, Yitong Yang, Meng Zhao, Mingzhu Yang, Zhaoling Shi

**Affiliations:** 1 Shaanxi University of Chinese Medicine, 712000 Xianyang, Shaanxi China; 2 The Second Affiliated Hospital of Shaanxi, University of Chinese Medicine, 712000 Xianyang, Shaanxi China; 3 Department of Pediatric Internal Medicine, Xian Yang Central Hospital, 712000 Xianyang, China

**Keywords:** Kawasaki disease, Caregivers, Knowledge, Attitudes, Practices, Cross-sectional study

## Abstract

**Purpose:**

To examine the knowledge, attitudes, and practices (KAP) of caregivers of children with Kawasaki disease toward Kawasaki disease.

**Methods:**

This cross-sectional study was conducted at four hospitals in China from March 2023 to June 2023. The KAP scores were evaluated using a self-designed questionnaire (Cronbach’s α = 0.840; KMO = 0.7381). Correlations between dimension scores were evaluated by Pearson correlation analysis. A structural equation model (SEM) was used to examine the relationships among factors.

**Results:**

Of 643 surveyed, 49.50% were male caregivers. The mean knowledge, attitude, and practice scores were 7.12 ± 2.34 (possible range, 0–11), 29.23 ± 5.67 (possible range, 12–60), and 21.57 ± 5.34 (possible range, 6–30). Knowledge correlated with attitude (*r* = 0.172, *P* < 0.001) and practice (*r* = 0.280, *P* < 0.001). Attitude was significantly related to practice (*r* = 0.598, *P* < 0.001). SEM showed knowledge had a positive effect on attitudes (β = 0.581, *P* < 0.001) and practices (β = 0.786, *P* < 0.001). In addition, attitudes also positively affected practices (β = 0.554, *P* < 0.001). Occupation type (β = 0.598, *P* = 0.025) and monthly per capita income (β=-0.750, *P* = 0.020) had different effects on attitudes, while monthly per capita income also had negative effects on practices (β=-0.410, *P* = 0.021).

**Conclusion:**

Caregivers of children with Kawasaki disease have moderate knowledge and unfavorable attitudes but proactive practices toward this disease. The results could help design an educational intervention to improve KAP, which could translate into better patient management and outcomes.

**Trial registration:**

Not applicable.

**Supplementary Information:**

The online version contains supplementary material available at 10.1186/s12889-024-18407-y.

## Background

Kawasaki disease is a self-limited, acute systemic vasculitis of unknown etiology [1, 2]. In the United States of America (USA), the incidence of Kawasaki disease is 23.9 per 100,000 children under 1 year old [3], while in Japan, the incidence is 308 per 100,000 children aged 0–4 years [4]. Notably, this condition appears to be more prevalent among Japanese and black children [2, 5], affecting predominantly those under 5 years of age (75-85% of cases) [1, 2]. The management of acute Kawasaki disease focuses on controlling inflammation and preventing arterial damage and thrombosis until systemic inflammation resolves and coronary artery luminal dimensions stabilize [1, 6]. Kawasaki disease stands as the primary cause of acquired heart disease in children in developed countries [1, 2]. Approximately 25% of children diagnosed with Kawasaki disease develop coronary artery dilatations or aneurysms [7, 8].

The long-term management of Kawasaki disease encompasses patient surveillance, thromboprophylaxis, and the prevention of myocardial ischemia resulting from coronary artery thrombosis, stenosis, or occlusions. Given that the management of Kawasaki disease involves acetylsalicylic acid (ASA), caregivers of children undergoing chronic ASA therapy should be advised to promptly contact their child’s physician if the child exhibits symptoms of or is exposed to either influenza or varicella [1].

Numerous studies have indicated that caregivers harbor enduring concerns about their child’s health post-Kawasaki disease, irrespective of their child’s coronary artery status [9–11]. As Kawasaki disease typically manifests in early childhood, primary educational efforts are directed toward caregivers, recognizing them as pivotal figures in patient self-management [12]. Knowledge about the symptoms, the importance of consulting promptly, and primary management of the child should help reach the most appropriate outcomes. Given that Kawasaki disease occurs in young children ≤ 5 years old, caregivers’ knowledge, attitudes, and practices (KAP) toward Kawasaki disease are crucial to managing the condition. KAP surveys provide quantitative and qualitative data about gaps in knowledge, misunderstandings, and misconceptions that could hinder the performance of a specific subject in a specific population [13, 14]. KAP surveys provide information for designing educational interventions to improve KAP toward a specific subject and improve public health. The KAP theory suggests that knowledge is the basis for changes in behaviors, and attitudes are the driving force for the changes [13, 14]. Besides the studies that reported worries for their children after Kawasaki disease [9–11], no previous studies are available about the KAP toward Kawasaki disease in the caregivers of children with Kawasaki disease. One study reported variable KAP toward fever among caregivers of young children, including one knowledge question regarding the possibility of Kawasaki disease in children with fever lasting more than 5 days [15]. Another study reported that the increasing prevalence of Kawasaki disease in India could, in fact, be due to a higher awareness of the disease [16]. Even among pediatric cardiologists in the USA, the KAP toward Kawasaki disease appears variable [17]. Therefore, it is important to carry out KAP studies among the caregivers of children with Kawasaki disease to be able to target defective knowledge and inappropriate attitudes and ensure the most optimal practice.

Considering the major lack of gap regarding the KAP toward Kawasaki disease, this study aimed to examine the KAP of caregivers of children with Kawasaki disease toward Kawasaki disease. The results could help determine the topics that require improvements and design appropriate educational tools and interventions.

## Materials and methods

This study was reported according to the STROBE statement [18].

### Study design and participants

This cross-sectional study was a multicenter study conducted at the Second Affiliated Hospital of Shaanxi University of Chinese Medicine, Caihong Hospital of Xianyang City, Xianyang Central Hospital, and Xi’ an International Medical Center from March 2023 to Jun 2023. This study was approved by the Medical Ethics Committee of the Second Affiliated Hospital of Shaanxi University of Chinese Medicine (approval #BA2023002) as the lead center, as well as by the ethical committees of all participating centers. All participants signed the informed consent form before participating in the study. The questionnaires did not contain any identifying information and were identified by a sequential participant number. The correspondence key to the actual participant identity was kept in a separate encrypted file only to ensure that a given participant would not be enrolled twice. The correspondence key was destroyed once the study was completed. All data are kept on a secure server and were anonymized.

The participants were caregivers of children with Kawasaki disease. The inclusion criteria were (1) the participant’s child had a diagnosis of Kawasaki disease that met the 2017 criteria by the American Heart Association [1], (2) the primary caregiver of the child with Kawasaki disease, and (3) caregivers who voluntarily participated in the questionnaire survey. The exclusion criteria were caregivers with cognitive impairment or mental illness.

### Sample size

The minimum required sample size was calculated using the sample size calculation formula:

$$ n=\left(\frac{{Z}_{1}-a/2}{\delta }\right)\times p\times \left(1-p\right)$$

where p was assumed as 0.5, δ as 0.05, and α = 0.05. The final plan entails collecting a minimum of 480 questionnaires, considering an anticipated effective response rate of 80%.

### Procedures

The questionnaire was designed based on the AHA Scientific Statement on Kawasaki Disease (2017) [1], other guidelines [6, 19], and the literature [16, 17]. The first draft of the questionnaire was revised based on the opinions of three senior experts. After that, 85 questionnaires were collected to carry out a pilot study. The reliability and validity test showed Cronbach’s α = 0.840, indicating high internal consistency. The KMO value was 0.7381, and the sampling quantity was acceptable.

The final questionnaire was in Chinese and included four aspects: demographic data (gender, age, guardian type, daily caregiver, occupation type, monthly family income, educational level, number of hospital admissions, gender of child, age of child, and number of follow-ups of the child), knowledge dimension, attitude dimension, and practice dimension. The knowledge dimension included 11 questions in two aspects. One point was scored for correct answers, and zero points were scored for wrong or unclear answers. The score range was 0–11 points. The attitudes dimension consisted of 12 questions using a 5-point Likert scale from very positive (5 points) to very negative (1 point). The score range was 12–60 points. The practices dimension included 11 questions. Among them, six questions used the 5-point Likert scale from always (5 points) to never (1 point). The score range was 6–30 points. The remaining five practice questions were open-ended and unscored and were used to assess whether the participants had participated in genetic screening for genetic predisposition related to Kawasaki disease, how they learned about Kawasaki disease, physical and psychological problems they had in caring for the child, and possible problems, they had major concerns about during treatment. A score above 70% of the total score is considered good, a score between 50% and 70% is considered moderate, and a score below 50% is considered poor.

The electronic questionnaires were distributed via the QQ (Tencent, China) group established by caregivers of children with Kawasaki disease using a link created by Questionnaire Star (Changsha Ranxing Information Technology Co., Ltd), an online questionnaire software platform. The use of IP address tracking and mandatory completion of all questionnaire items helped to ensure the validity and reliability of the data collected. The research team checked all questionnaires for completeness, internal coherence, and reasonableness.

### Statistical analysis

Stata 17.0 (Stata Corporation, College Station, TX, USA) was used for analysis. Demographic data and KAP scores were analyzed descriptively, and data were expressed as means ± standard deviations after confirming the normal distribution using the Kolmogorov-Smirnov test. The differences in knowledge, attitude, and practice scores of participants with different demographic characteristics were compared by the Kruskal-Wallis equality-of-populations rank test. The categorical data were expressed as n (%) and analyzed using the chi-squared test. Spearman correlation analysis was used to evaluate the correlation among KAP dimensions. A structural equation model (SEM) was used to test the hypotheses that (1) knowledge affects attitude, (2) knowledge affects practice, (3) knowledge also affects practice through attitude, and (4) attitude directly affects practice. Two-sided *P*-values < 0.05 were considered statistically significant.

## Results

### Characteristics of the participants

A total of 643 questionnaires were distributed, and 643 valid questionnaires were received. The valid questionnaire recovery rate was 100%. Most participants were female (50.50%), 26–35 years of age (57.90%), the parents of the child (79.20%), parents as the regular caregiver of the child (86.60%), working in commercial and service industry (43.20%), income of 10,000–20,000 (58.20%), and undergraduate degree (81.60%). Hence, the participants were mostly of a high socioeconomic status. The children were mostly male (55.20%), 3–5 years of age (40.30%), with at least two admissions for Kawasaki disease (47.10%), and with at least two follow-ups after treatment (Table 1).

**Table 1:** Demographic information

Variables	n (%)	Knowledge scores		Attitude scores		Practice scores	
		Mean ± SD	P	Mean ± SD	P	Mean ± SD	P
**Total scores**	643	7.12 ± 2.34		29.23 ± 5.67		21.57 ± 5.34	
**Gender**			0.205		0.280		0.091
Male	318 (49.50)	7.20 ± 2.33		29.02 ± 5.62		21.92 ± 5.23	
Female	325 (50.50)	7.04 ± 2.35		29.43 ± 5.72		21.22 ± 5.44	
**Age**			0.904		0.417		0.440
18–25	318 39 (6.10)	7.18 ± 2.32		28.95 ± 5.92		22.46 ± 5.51	
26–35	372 (57.90)	7.06 ± 2.39		29.20 ± 5.76		21.59 ± 5.45	
36–40	141 (21.90)	7.20 ± 2.38		28.87 ± 5.47		21.06 ± 5.06	
> 40	91 (14.20)	7.22 ± 2.13		29.99 ± 5.54		21.87 ± 5.25	
**Relationship with child**			0.229		0.004		0.833
Parents	509 (79.20)	7.10 ± 2.38		29.32 ± 5.76		21.59 ± 5.49	
Grandparents/great-grandparents	124 (19.30)	7.22 ± 2.23		28.50 ± 5.23		21.48 ± 4.70	
Other	10 (1.60)	6.60 ± 1.35		33.40 ± 4.67		21.40 ± 5.74	
**Regular caregiver for the child**			0.818		0.200		0.571
Parents	557 (86.60)	7.13 ± 2.34		29.08 ± 5.69		21.49 ± 5.38	
Grandparents/great-grandparents	66 (10.30)	7.05 ± 2.50		29.83 ± 5.50		21.77 ± 5.24	
Aunt/nanny	9 (1.40)	6.78 ± 2.49		28.33 ± 5.10		23.33 ± 5.32	
Other	11 (1.70)	7.09 ± 1.58		33.55 ± 4.66		22.64 ± 4.13	
**Occupation type**			0.048		< 0.0001		0.098
Responsible persons of party and mass organizations, enterprises, and institutions of state organs	121 (18.80)	6.93 ± 2.49		27.86 ± 5.63		21.12 ± 5.06	
Professional and technical personnel (teachers, doctors, engineering technicians, writers, etc.)	178 (27.70)	7.46 ± 2.17		29.64 ± 5.41		22.37 ± 5.12	
Commercial and service industry personnel	278 (43.20)	7.05 ± 2.38		28.81 ± 5.83		21.44 ± 5.47	
Other	66 (10.30)	6.82 ± 2.29		32.35 ± 4.51		20.80 ± 5.74	
**Monthly per capita income**			0.680		< 0.0001		0.803
< 2000	10 (1.60)	7.50 ± 1.27		33.30 ± 3.37		22.20 ± 4.64	
2000–5000	59 (9.20)	6.83 ± 2.46		32.19 ± 4.79		21.66 ± 5.77	
5000-10,000	160 (24.90)	7.22 ± 2.17		29.15 ± 5.42		21.43 ± 5.38	
10,000–20,000	374 (58.20)	7.07 ± 2.44		28.63 ± 5.76		21.50 ± 5.29	
> 20,000	40 (6.20)	7.48 ± 2.12		29.75 ± 5.85		22.45 ± 5.40	
**Educational level**			0.874		0.004		0.885
Junior high school and below	28 (4.40)	7.43 ± 2.18		32.25 ± 5.27		21.29 ± 6.27	
High school and secondary school	47 (7.30)	6.89 ± 2.56		30.32 ± 5.35		21.13 ± 5.84	
Undergraduate and tertiary degrees	525 (81.60)	7.12 ± 2.33		28.93 ± 5.62		21.68 ± 5.24	
Graduate degree or above	43 (6.70)	7.14 ± 2.44		29.65 ± 6.35		20.86 ± 5.49	
**Number of hospital admissions for Kawasaki disease**			0.721		0.101		0.478
1	241 (37.50)	7.04 ± 2.37		29.31 ± 5.80		21.40 ± 5.71	
≥ 2	303 (47.10)	7.12 ± 2.37		28.86 ± 5.55		21.83 ± 5.11	
0	99 (15.40)	7.31 ± 2.19		30.15 ± 5.69		21.18 ± 5.12	
**Gender of child**			0.877		0.206		0.810
Male	355 (55.20)	7.09 ± 2.34		29.03 ± 5.68		21.62 ± 5.38	
Female	288 (44.80)	7.15 ± 2.35		29.47 ± 5.67		21.50 ± 5.31	
**Age of child**			0.662		0.748		0.297
0–2 years old	217 (33.70)	7.14 ± 2.29		29.48 ± 5.56		21.67 ± 5.16	
3–5 years old	259 (40.30)	7.03 ± 2.40		28.93 ± 5.92		21.13 ± 5.52	
≥ 6 years old	167 (26.00)	7.22 ± 2.32		29.35 ± 5.44		22.11 ± 5.27	
**Number of follow-ups of the child after treatment**			0.052		0.008		0.467
1	180 (28.00)	7.38 ± 2.29		29.15 ± 5.59		21.96 ± 5.04	
≥ 2	361 (56.10)	7.05 ± 2.37		28.90 ± 5.57		21.50 ± 5.40	
0	102 (15.90)	6.91 ± 2.30		30.52 ± 6.05		21.13 ± 5.68	

### Knowledge

The mean knowledge scores were 7.12 ± 2.34 (possible range: 0–11) (64.73%), indicating moderate knowledge. High knowledge scores were found in professional and technical personnel (*P* = 0.048) (Table 1), suggesting a higher socioeconomic status. The knowledge item with the highest rate of correct answers was K5 (81.49%, “In the acute stage of Kawasaki disease, intravenous gamma globulin can be injected for emergency treatment.”), while the item with the lowest rate of correct answers was K6 (15.86%, “In the acute stage of Kawasaki disease, oral aspirin is also required to treat rash and fever.”) (Supplementary Table 1).

### Attitude

The mean attitude scores were 29.23 ± 5.67 (possible range: 12–60) (48.72%), indicating unfavorable attitudes. Higher attitude scores were observed in parents (*P* = 0.004), professional and technical personnel (*P* < 0.0001), with lower income (*P* < 0.0001), with lower educational level (*P* = 0.004), and with no follow-up after treatment (*P* = 0.008) (Table 1). Supplementary Table 2 shows the distribution of the attitudes toward the attitude items.

### Practice

The mean practice scores were 21.57 ± 5.34 (possible range: 6–30) (71.90%), suggesting proactive practice. No variables showed differences in practice scores (Table 1). Supplementary Table 3 shows the distribution of the habits toward the practice items. The most important source of information for the caregivers was public media, followed by medical staff and relevant training. Most participants felt tired and sick when helping their children recover. Most participants felt angry and were worried about their child’s future. The most important source of information about Kawasaki disease was pubic media (88.96%), followed by healthcare providers 69.36%), other parents of children with Kawasaki disease (67.96%), relevant training (62.99%), and literature and research reports (37.95%). When caring for their children, the patients felt tired during the day (86.00%), were feeling sick (80.72%), felt weak (74.18%), were waking up sleepy in the morning (69.98%), and were having headaches (37.64%). In addition, the parents felt helpless and hopeless (82.89%), felt angry (75.43%), felt sad (53.65%), felt anxious (48.06%), and felt very depressed (40.44%). During treatments, most parents were worried about their child’s future (94.25%), were worried about treatment effectiveness (91.91%), were worried about the impact of the disease on the other family members (61.59%), were worried about others’ reaction to the disease (58.79%), and were worried about the treatment side effects (42.77%).

### Correlations

Table 2 shows that the knowledge scores were correlated with the attitude (*r* = 0.172, *P* < 0.001) and practice (*r* = 0.280, *P* < 0.001) scores. The attitude scores were correlated to the practice scores (*r* = 0.598 *P* < 0.001).

**Table 2:** Correlation analysis among knowledge, attitudes and practices

	Knowledge	Attitudes	Practices
Knowledge	1		
Attitudes	0.172 (*p* < 0.001)	1	
Practices	0.280 (*p* < 0.001)	0.598 (*p* < 0.001)	1

### Structural equation modeling

**Fig. 1:**
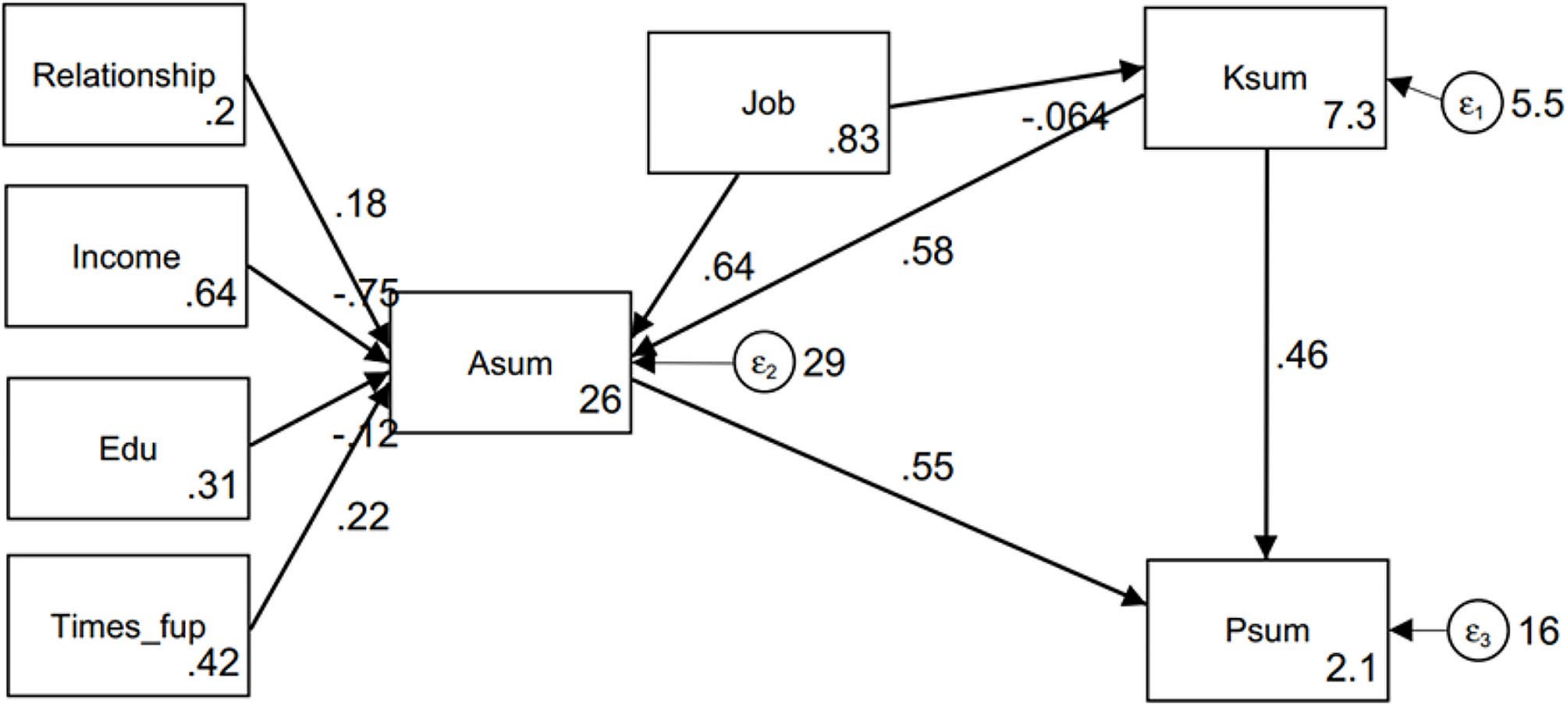
Structural equation model on KAP of caregivers of children with Kawasaki disease based on the theory of planned behavior. Standardized path coefficients were presented. Ksum: Knowledge, Asum: Attitudes, Psum: Practices, Job: Occupation type, Relationship: Relationship with child, Income: Monthly per capita income, Edu: Educational level, Times_fup: Number of follow-ups of the child after treatment

Figure 1 presents the schematic of the SEM. Knowledge had a positive effect on attitudes (β = 0.581, *P* < 0.001) and practices (β = 0.786, *P* < 0.001). In addition, attitudes also positively affected practices (β = 0.554, *P* < 0.001). Occupation type (β = 0.598, *P* = 0.025) and monthly per capita income (β=-0.750, *P* = 0.020) had different effects on attitudes, while monthly per capita income also had negative effects on practices (β=-0.410, *P* = 0.021). Other variables, including relationship with the child, educational level, and number of follow-ups of the child after treatment, did not influence knowledge, attitudes, or practices (Supplementary Table 4). The goodness-of-fit was acceptable to good depending upon the model used (Supplementary Table 5).

## Discussion

No previous studies examined the KAP toward Kawasaki disease of caregivers of children with Kawasaki disease. Considering the major lack of gap regarding the KAP toward Kawasaki disease, this study aimed to examine the KAP of caregivers of children with Kawasaki disease toward Kawasaki disease. This study showed that the caregivers of children with Kawasaki disease have moderate knowledge and unfavorable attitudes but proactive practices towards Kawasaki disease. Knowledge had a direct effect on attitudes and practices. Knowledge also had an indirect effect on practices. Attitude had a direct effect on practices. Income had a direct effect on practices. The number of admissions for Kawasaki disease did not influence the KAP dimensions. Educational level did not influence practices. Hence, improving knowledge might improve attitudes and practices.

Since Kawasaki disease occurs in infants and young children, the concept of “self-management” must be enlarged to include the caregivers. Therefore, most of the early education is aimed at the caregivers. According to the available guidelines, knowledge goals should include (1) specifics of Kawasaki disease history and complications, including cardiac events and procedures with dates or ages, (2) the importance of uninterrupted life-long cardiology care, (3) names, doses, and reasons for taking all medications, and the requirements for monitoring for specific drugs, (4) names and reasons for the tests performed, (5) specific symptoms or signs that warrant immediate medical attention, (6) recommendations regarding physical activity, (7) considerations regarding contraception, pregnancy, and recurrence of Kawasaki disease in offspring, (8) expectations regarding long-term prognosis and health, and (9) importance of and strategies to achieve healthy lifestyle behaviors [12].

Those points above were covered in the present study, showing that the caregivers of children with Kawasaki disease had moderate knowledge regarding Kawasaki disease and its management. No previous similar KAP studies are available in the literature for comparison, except maybe a study that showed variable KAP toward fever in children, including fever lasting > 5 days [15]. Indeed, a fever that lasts several days and responds poorly to the usual over-the-counter fever management drugs and methods should be considered suspicious for Kawasaki disease and should prompt immediate consultation [1, 6, 19]. The present study revealed that gaps in knowledge toward Kawasaki disease could be observed in the conditions associated with Kawasaki disease, the symptoms and signs of Kawasaki disease, the diagnostic process of Kawasaki disease, the role of aspirin, and the possibility of exercise in the children. Another study did not evaluate the KAP but nevertheless reported that increasing awareness of Kawasaki disease could be responsible for the increasing prevalence of Kawasaki disease observed in India over recent years [16]. The present study did not evaluate the KAP of healthcare providers, but a study in the USA reported that the KAP toward Kawasaki disease should be improved among pediatric cardiologists [17]. Indeed, healthcare providers are major sources of reliable health-related information for the general population [20]. Future studies should examine their KAP toward Kawasaki disease and whether continuing education activities would be necessary. Improving the KAP of healthcare providers could help improve the KAP of the caregivers of children with Kawasaki disease. Still, the present study indicates that educational and motivational interventions should be designed to improve the KAP of the population toward Kawasaki disease. Indeed, the present study was performed in caregivers of children with Kawasaki disease. Hence, the caregivers discussed with healthcare providers and received some information about the disease. Still, their knowledge level was moderate at best, hinting that the knowledge level of the general population could be even lower. It should also be examined in future studies.

In the present study, the attitude was unfavorable, but the practice was proactive, suggesting that the caregivers generally followed the physician’s advice and instructions without understanding them or actively agreeing with them. Still, according to the KAP theory, knowledge is the basis for changes in behaviors, and attitudes are the driving force for the changes [13, 14]. Therefore, improvements in knowledge should translate into improvements in attitudes, which would lead to more proactive practices. It is supported by the SEM analysis, which showed such relationships in the present study.

The SEM analysis showed that income influenced practices. As patients pay for their healthcare services in China, a better socioeconomic status might be associated with better practices and proactive consultations. A better socioeconomic status is also associated with better health literacy (i.e., knowledge) [21], and the SEM analysis showed that knowledge, directly and indirectly, influenced practice. Most participants felt angry about their child’s condition and were worried about their child’s future, which has been reported in the literature [9–11]. On the other hand, the number of hospital admissions for Kawasaki disease had no influence on the KAP dimensions. It could be because the parents trust the medical teams to take care of their child, they fear interfering with the medical team’s work if they are asking too many questions, or they feel too helpless to gain knowledge. Additional studies could examine that.

This study had limitations. Even though four centers participated in the study, the number of participants might appear limited, but considering that Kawasaki disease is rare, the sample size could, in fact, be considerable. Nevertheless, all participants were from the same geographical area, limiting the generalizability of the study. A nationwide study could help determine the actual situation of KAP toward Kawasaki disease in China, especially considering the disparities in socioeconomic statuses among provinces. The questionnaire was designed by local investigators, and it is possible that local guidelines, clinical habits, and policies biased the elaboration of the questionnaire, also limiting generalizability. All KAP studies are at risk of the social desirability bias, in which the participants can be tempted to answer what they should do instead of what they do [22, 23]. On the other hand, considering that the knowledge was low, the likelihood of the social desirability bias is low. Finally, KAP studies are cross-sectional and represent only a snapshot of a precise situation in time. Nevertheless, the present study could serve as a historical baseline for future studies evaluating the impact of educational intervention on Kawasaki disease.

## Conslusions

In conclusion, the caregivers of children with Kawasaki disease have moderate knowledge and unfavorable attitudes but proactive practices toward this disease. The results could help design an educational intervention to improve KAP, which could translate into better patient management and outcomes.

### Electronic supplementary material

Below is the link to the electronic supplementary material.

Supplementary Material 1

## Data Availability

All data generated or analysed during this study are included in this published article [and its supplementary information files].

## Abbreviations

KAP: Knowledge, attitudes, and practices
SEM: Structural equation model
ASA: Acetylsalicylic acid
