# Supplementary material for: Knowledge, attitudes, and practices towards Kawasaki disease from caregivers of children with Kawasaki disease: a cross-sectional study
Source: BMC Public Health. 2024 Mar 26;24:899. doi: 10.1186/s12889-024-18407-y (PMC10967092; doi:10.1186/s12889-024-18407-y)
Supplement: Supplementary file 1 — Supplementary Material 1 [file 12889_2024_18407_MOESM1_ESM.docx]

**Supple**

**mentary Table 1.** Knowledge dimension of caregivers of children with Kawasaki disease

|  | True | False | Unclear |
| --- | --- | --- | --- |
| 1. Kawasaki disease, also known as mucocutaneous lymph node syndrome, mainly causes rash, lymphadenopathy, and other reactions and does not cause cardiovascular damage. (correct answer: False) | 146 (22.71) | 428 (66.56) | 69 (10.73) |
| 2. Kawasaki disease may occur due to bacterial or viral infections. (correct answer: True) | 218 (33.9) | 372 (57.85) | 53 (8.24) |
| 3. Kawasaki disease is genetically sensitive, and certain genotype mutations in children can cause susceptibility to Kawasaki disease. (correct answer: True) | 465 (72.32) | 91 (14.15) | 87 (13.53) |
| 4. Kawasaki disease can be detected by blood tests. (correct answer: False) | 150 (23.33) | 449 (69.83) | 44 (6.84) |
| 5. In the acute stage of Kawasaki disease, intravenous gamma globulin can be injected for emergency treatment. (correct answer: True) | 524 (81.49) | 78 (12.13) | 41 (6.38) |
| 6. In the acute stage of Kawasaki disease, oral aspirin is also required to treat rash and fever. (correct answer: False) | 468 (72.78) | 102 (15.86) | 73 (11.35) |
| 7. During mouth ulcers in children with Kawasaki disease, it is suitable for a nutrient-rich and easily digestible liquid or semi-liquid diet. (correct answer: True) | 480 (74.65) | 94 (14.62) | 69 (10.73) |
| 8. Children with Kawasaki disease can exercise moderately during acute fever to maintain their mental condition. (correct answer: False) | 93 (14.46) | 408 (63.45) | 142 (22.08) |
| 9. After discharge due to recovering from the acute stage of Kawasaki disease, it is necessary to follow up and re-examine within 8-12 weeks to confirm that the acute stage of Kawasaki disease has been safely passed. (correct answer: True) | 518 (80.56) | 88 (13.69) | 37 (5.75) |
| 10. After the acute phase of Kawasaki disease, follow-up should still be performed every 3-6 months. (correct answer: True) | 468 (72.78) | 103 (16.02) | 72 (11.2) |
| 11. Parents of children with Kawasaki disease need to pay attention to their mental health, and good emotions can help their children recover as soon as possible. (correct answer: True) | 517 (80.4) | 83 (12.91) | 43 (6.69) |

**Supplementary Table 2.** Attitudes dimension of caregivers of children with Kawasaki disease

|  | Strongly agree | Agree | Neutrality | Disagree | Strongly disagree |
| --- | --- | --- | --- | --- | --- |
| 1. You think it is necessary to learn about Kawasaki disease by reading relevant literature and popularizing science. | 244 (37.95) | 233 (36.24) | 102 (15.86) | 53 (8.24) | 11 (1.71) |
| 2. You think it is necessary to learn how to handle Kawasaki disease and how to deal with it in an emergency. | 227 (35.3) | 207 (32.19) | 105 (16.33) | 69 (10.73) | 35 (5.44) |
| 3. You think that discussing Kawasaki disease with your healthcare providers in time will greatly help you improve your knowledge. | 254 (39.5) | 198 (30.79) | 105 (16.33) | 53 (8.24) | 33 (5.13) |
| 4. You believe that Kawasaki disease is very serious, and mishandling can have a huge impact on a child’s life. | 218 (33.9) | 207 (32.19) | 117 (18.2) | 70 (10.89) | 31 (4.82) |
| 1. You think the child should seek medical attention in time if there is a high fever, rash, eyeball congestion, edema of hands and feet, etc. | 244 (37.95) | 203 (31.57) | 99 (15.4) | 56 (8.71) | 41 (6.38) |
| 6. You think you are confident that you can do a good job of emergency care on a child with acute stage Kawasaki disease. | 210 (32.66) | 197 (30.64) | 123 (19.13) | 69 (10.73) | 44 (6.84) |
| 7. You are confident you can give your child medication exactly as prescribed. | 232 (36.08) | 197 (30.64) | 103 (16.02) | 69 (10.73) | 42 (6.53) |
| 8. You trust the pediatrician who gives the treatment. | 228 (35.46) | 220 (34.21) | 99 (15.4) | 64 (9.95) | 32 (4.98) |
| 9. You trust the efficacy of your treatment plan. | 241 (37.48) | 206 (32.04) | 108 (16.8) | 61 (9.49) | 27 (4.2) |
| 10. You believe that the relevant examination proposed by the doctor is necessary to diagnose Kawasaki disease. | 230 (35.77) | 198 (30.79) | 115 (17.88) | 55 (8.55) | 45 (7) |
| 11. You believe your child should have regular follow-ups for cardiac ultrasound even after discharge from Kawasaki disease. | 242 (37.64) | 201 (31.26) | 97 (15.09) | 69 (10.73) | 34 (5.29) |
| 12. You think your child with Kawasaki disease makes you very worried and scared. | 211 (32.81) | 223 (34.68) | 112 (17.42) | 64 (9.95) | 33 (5.13) |

**Supplementary Table 3.** Practices dimension of caregivers of children with Kawasaki disease

|  | Always | Usually | Sometimes | Occasionally | Never |  |
| --- | --- | --- | --- | --- | --- | --- |
| 1. How often do you learn about Kawasaki disease? | 140 (21.77) | 208 (32.35) | 136 (21.15) | 105 (16.33) | 54 (8.4) |  |
| 2. How often do you communicate with medical staff about daily care related to Kawasaki disease, emergency care methods, etc? | 164 (25.51) | 190 (29.55) | 141 (21.93) | 99 (15.4) | 49 (7.62) |  |
| 3. You control your child’s daily diet and exercise | 166 (25.82) | 222 (34.53) | 129 (20.06) | 75 (11.66) | 51 (7.93) |  |
| 4. You regularly take your child to the hospital for follow-up visits to review Kawasaki disease. | 193 (30.02) | 202 (31.42) | 131 (20.37) | 74 (11.51) | 43 (6.69) |  |
| 5. You can successfully provide appropriate emergency care to children with acute Kawasaki disease based on scientific methods. | 201 (31.26) | 204 (31.73) | 126 (19.6) | 76 (11.82) | 36 (5.6) |  |
| 6. You are willing to pass on your knowledge about Kawasaki disease to other relatives or patients. | 200 (31.1) | 191 (29.7) | 129 (20.06) | 90 (14) | 33 (5.13) |  |
|  | Yes | No |  |  |  |  |
| 7. Have you participated in screening for genetic susceptibility genotypes associated with Kawasaki disease? | 278 (43.23) | 365 (56.77) |  |  |  |  |
|  | Literature and research reports | Public media | Medical staff | Parents of other Kawasaki disease children | Participate in relevant training | Others |
| 8. How do you learn about Kawasaki disease? (multiple options) | 244 | 572 | 446 | 437 | 405 | 1 |
|  | I feel tired during the day | I wake up sleepy in the morning | I feel a headache | I feel weak | I feel sick to my stomach | Others |
| 9.1 Have you had any physical problems during helping your child recover? (multiple options) | 553 | 450 | 242 | 477 | 519 | 2 |
|  | I feel anxious | I feel sad | I feel angry | I feel very depressed | I feel helpless and hopeless | Others |
| 9.2 Have you had any emotional problems during helping your child recover? (multiple options) | 309 | 345 | 485 | 260 | 533 | 0 |
|  | I’m worried about the effectiveness of the treatment | I’m worried about the side effects of the treatment | I’m worried about how people will react to the child’s disease | I’m worried about how the disease will affect other family members | I’m worried about my child’s future | Others |
| 10. Because of your child’s health condition, what are your concerns about during the entire treatment? (multiple options) | 591 | 275 | 378 | 396 | 606 | 1 |

**Supplementary Table 4.** Parameters of the structural equation model.

| **Model paths** | | | **Total effects** | | **Direct Effect** | | **Indirect effect** | |
| --- | --- | --- | --- | --- | --- | --- | --- | --- |
|  | | | β (95% CI) | P | β (95% CI) | P | β (95% CI) | P |
| Ksum | <- | Job | -0.06(-0.26,0.13) | 0.527 | -0.06(-0.26,0.13) | 0.527 | - | - |
| Asum | <- | Ksum | 0.581(0.402,0.76) | <0.001 | 0.581(0.402,0.76) | <0.001 | - | - |
| Asum | <- | Relationship | 0.178(-0.76,1.12) | 0.713 | 0.178(-0.76,1.12) | 0.713 | - | - |
| Asum | <- | Job | 0.598(0.074,1.12) | 0.025 | 0.635(0.124,1.14) | 0.015 | -0.03(-0.15,0.07) | 0.529 |
| Asum | <- | Income | -0.75(-1.38,-0.11) | 0.02 | -0.75(-1.38,-0.11) | 0.02 | - | - |
| Asum | <- | Edu | -0.12(-1.01,0.76) | 0.784 | -0.12(-1.01,0.76) | 0.784 | - | - |
| Asum | <- | Times_fup | 0.217(-0.46,0.89) | 0.532 | 0.217(-0.46,0.89) | 0.532 |  | - |
| Psum | <- | Ksum | 0.786(0.618,0.95) | <0.001 | 0.463(0.328,0.59) | <0.001 | 0.322(0.223,0.42) | <0.001 |
| Psum | <- | Asum | 0.554(0.498,0.61) | <0.001 | 0.554(0.498,0.61) | <0.001 | - | - |
| Psum | <- | Relationship | 0.098(-0.42,0.62) | 0.713 | - | - | 0.098(-0.42,0.62) | 0.713 |
| Psum | <- | Job | 0.302(-0.02,0.62) | 0.069 | - | - | 0.302(-0.02,0.62) | 0.069 |
| Psum | <- | Income | -0.41(-0.77,-0.06) | 0.021 | - | - | -0.41(-0.77,-0.06) | 0.021 |
| Psum | <- | Edu | -0.06(-0.56,0.42) | 0.784 | - | - | -0.06(-0.56,0.42) | 0.784 |
| Psum | <- | Times_fup | 0.120(-0.25,0.49) | 0.532 | - | - | 0.120(-0.25,0.49) | 0.532 |

Ksum: Knowledge, Asum: Attitudes, Psum: Practices, Job: Occupation type, Relationship: Relationship with child, Income: Monthly per capita income, Edu: Educational level, Times_fup: Number of follow-ups of the child after treatment

**Supplementary Table 5.** Goodness-of-fit of the structural equation mode.

|  | **Value** | **Indicate** |
| --- | --- | --- |
| RMSEA | 0.050 | Acceptable |
| CFI | 0.967 | Good fit |
| TLI | 0.935 | Acceptable |
| SRMR | 0.029 | Good fit |
